# Intraocular Lymphoma: A Review

**DOI:** 10.18502/jovr.v20.17181

**Published:** 2025-12-08

**Authors:** Saeed Karimi, Kimia Daneshvar

**Affiliations:** Ophthalmic Research Center, Research Institute for Ophthalmology and Vision Science, Shahid Beheshti University of Medical Sciences, Tehran, Iran

**Keywords:** Intraocular Lymphoma, Masquerade Syndromes, Uveal Lymphoma, Vitreoretinal Lymphoma

## Abstract

Intraocular lymphoma (IOL) is a rare form of non-Hodgkin lymphoma that primarily presents in two distinct types. The first type, known as primary intraocular lymphoma (PIOL), is mainly recognized as a subtype of primary central nervous system lymphoma (PCNSL). Recent classifications have emphasized the primary ocular sites affected, with vitreoretinal lymphoma emerging as the most common variant linked to PCNSL. Despite its rarity, the incidence of PIOL is rising among both immunocompromised and immunocompetent populations. Most cases of PIOL are identified as diffuse large B-cell lymphoma, although rare T-cell variants have also been reported. Secondary intraocular lymphoma (SIOL) originates from metastatic spread of non-CNS lymphomas to the ocular structures, including the retina, uvea, vitreous body, Bruch's membrane, and optic nerve. Diagnosis of IOL is challenging for ophthalmologists and pathologists, as it can easily mimic other ocular conditions. Advancements in laboratory diagnostics, such as immunocytochemistry, flow cytometry, and the evaluation of interleukin ratios (specifically IL-10:IL-6 > 1), along with polymerase chain reaction (PCR) amplification for clonality, have enhanced diagnostic accuracy. Multimodal imaging approaches and molecular analyses can serve as valuable indicators of visual prognosis, recurrence rates, and the likelihood of progression to central nervous system involvement. Given that misdiagnosis or delayed diagnosis can result in serious treatment delays and potentially life-threatening outcomes for patients with IOL, this review seeks to provide a comprehensive understanding of the clinical manifestations of IOL and the diagnostic methods employed.

##  INTRODUCTION

Intraocular lymphoma (IOL) is a rare ophthalmic malignancy, with incidence rates ranging from 0.5 to 2.0 per million. IOL showed an increasing trend over the past few decades, reflecting improved diagnostic capabilities and greater awareness about the disease.^[1,2]^ The rising incidence of IOL in recent years can be linked to a growing number of patients with immunodeficiency and immunosuppression, increased life expectancy, and enhancements in diagnostic technologies. It typically affects older adults, with a predominance in females.^[3,4]^

IOL comprises a heterogeneous group of uncommon tumors that are often misdiagnosed and underrecognized, due to their insidious onset, low prevalence, and their tendency to masquerade as other ocular conditions. This rarity hinders clinical research and complicates the development of standardized management strategies.^[5,6]^ IOL can be categorized based on the specific intraocular structures affected, the origin of the lymphocytes (B- or T-lymphocytes), and whether the condition is primary, localized to the central nervous system (CNS), or secondary, stemming from systemic lymphoma. In terms of the specific structural involvement, IOL is classified into vitreoretinal lymphoma (VRL), which impacts the retina and vitreous body, and uveal lymphoma, which targets the iris, ciliary body, and choroid.^[7]^ More than 90% of cases are of B-cell origin and are commonly associated with primary CNS non-Hodgkin's lymphoma. In contrast, T-cell lymphomas are comparatively rare and mainly occur as secondary manifestations of metastatic systemic T-cell lymphomas.^[8,9]^

In patients with IOL, the incidence of CNS cases ranges from 60% to 80%.^[10]^ While 15% to 25% of individuals with primary central nervous system lymphoma (PCNSL) exhibit ophthalmic manifestations, a significantly higher proportion, between 56% and 90% of patients, with primary intraocular lymphoma (PIOL) will present with CNS manifestations of lymphoma, either at diagnosis or during the course of the disease.^[11,12]^

Given the rarity of IOLs, this review synthesizes the current literature on IOLs and their diagnosis, summarizing vital studies to highlight pitfalls and limitations, thereby enhancing clinical decision-making and identifying future challenges in the field.

##  METHODS 

A search was conducted on the online databases PubMed and Embase for publications from 1990 to April 2024 using the terms "intraocular lymphoma," "vitreoretinal lymphoma," "uveal lymphoma," "iris lymphoma," "choroidal lymphoma," and "ciliary body lymphoma." The inclusion criteria encompassed English-language articles discussing IOLs, including clinical research, non-clinical studies, meta-analyses, reviews, and case series. Non-English articles, case reports, or animal studies were excluded from the review.

##  RESULTS

### Primary Intraocular Lymphoma (PIOL)

PIOL is a subset of PCNSL that primarily affects the vitreoretinal region, commonly referred to as primary vitreoretinal lymphoma (PVRL). The median age of the patients was over 60 years,^[2,13]^ with an age range spanning from 15 to 85 years.^[6]^ PIOL predominantly affects females, with a female-to-male ratio of 2:1.^[14]^ The most common clinical symptoms and signs of PIOL include blurring of vision, observed in 72% of patients, and vitreous inflammatory opacity, noted in 92% of cases. The most common pathological type is diffuse large B-cell lymphoma (DLBCL); however, in patients under 60 years of age, extranodal marginal zone lymphoma (EMZL) of mucosa-associated lymphoid tissue is the most frequently observed type.^[2,15]^ The reported survival rates are 82% at 2 years and 70% at 5 years, with a recurrence rate of 44%. The CNS serves as the primary site for recurrence; while 19% of patients exhibit CNS involvement at the initial visit, this figure increases to 58% as the disease advances. Due to the wide variety of manifestations associated with PIOL, timely diagnosis has been significantly challenging, with a misdiagnosis rate of 64% and a delayed diagnosis rate of 85%.^[15]^

### Vitreoretinal Lymphoma (VRL)

#### Definition and manifestation 

VRLs predominantly originate from B-lymphocytes and are frequently linked to CNS lymphoma, with an incidence of 0.047 cases per 100,000 people per year.^[16]^ VRL is typically diagnosed in older individuals, specifically those aged 50 to 70. However, younger patients may develop VRL in the context of immunosuppression.^[17]^ While a minority of VRL arise from T-cells, these cases are generally linked to metastatic spread from systemic adult T-cell leukemia/lymphoma, categorizing them as secondary IOL.^[18]^ VRL typically presents with floaters and vision loss, along with distinct retinal findings such as multifocal creamy-white lesions and a "leopard-skin" appearance.^[19]^ They are highly aggressive malignant neoplasms that often present with bilateral ocular involvement, with the vitreous being affected in the majority of patients.^[20]^ It should be considered in immunosuppressed individuals of advanced age who present with vision changes that do not improve with steroid treatment.^[16,21]^

A minority of VRLs are secondary, arising from disseminated systemic lymphoma rather than being primary. Research indicates no significant differences in demographics, ocular findings, treatment responses, or visual outcomes between patients with PVRL and those with VRL associated with systemic lymphomas. However, it is noteworthy that CNS involvement is more prevalent in the group with VRL and systemic lymphoma following the onset of ocular symptoms.^[22]^

Younger individuals with VRL ( ≤ 50 years) exhibit unique clinical features, including a higher incidence of retinal and subretinal pigment epithelial infiltration and a lower *MYD88* mutation rate compared to older patients. They also experience a shorter time to CNS lymphoma onset. However, overall survival rates are similar between age groups. These findings highlight the importance of early diagnosis and close monitoring of young patients, given their unique disease characteristics.^[23]^

PVRL frequently presents a diagnostic challenge due to its heterogeneous manifestations. This complexity often leads to its classification as a masquerade syndrome, as it can mimic various ocular conditions, including uveitis,^[24,25,26,27]^ viral retinitis,^[28,29]^ age-related macular degeneration,^[30]^ endophthalmitis,^[31]^ Vogt-Koyanagi-Harada disease,^[32]^ occlusive retinal vasculitis,^[33]^ neovascular glaucoma,^[34]^ and central serous chorioretinopathy.^[35]^ Thus, a thorough evaluation of this disease and a careful investigation of diagnostic clues are crucial for timely diagnosis.

### Diagnosis 

#### Ocular fluid and tissue diagnostics

The misdiagnosis rate for PVRL has reached 100%, with 30% of cases incorrectly diagnosed as uveitis, significantly worsening the prognosis. This highlights the diagnostic challenges posed by PVRL's atypical signs and emphasizes the need for careful examination to avoid misidentification.^[36]^

The definitive method for diagnosing PVRL is vitreous biopsy. Vitreous biopsy, either through pars plana vitrectomy (PPV), preferably using the safer 25-gauge technique with up to 15,000 CPM,^[37]^ or vitreous aspiration, has been the primary diagnostic modality for VRL, relying on the identification of atypical lymphoid cells. However, this approach has inherent limitations,^[38]^ as the positive yield is approximately 50% due to the small volume of vitreous sample, scarcity and fragility of the lymphoma cells, as well as their potential sensitivity to prior steroid therapy. If patients are receiving systemic steroids for presumed uveitis, these medications should be discontinued at least 2 weeks prior to sampling to minimize the risk of false-negative results.^[39,40]^

Vitreous sampling is performed using a standard three-port PPV technique. The procedure begins with the collection of undiluted vitreous for cytological evaluation, followed by a diluted sample for cytokine analysis, which is obtained through an infuser. Additionally, any vitreous wash fluid collected in the vitrector cassette is used for microbiological cultures. To maintain cell viability, 1–2 mL of the vitreous should be placed in Roswell Park Memorial Institute (RPMI) culture medium, achieving a total fluid volume of 2–5 mL. Prompt transportation to a cytologist or ocular pathologist is crucial, as lymphoma cells can degenerate quickly. The sample undergoes centrifugation at 1000 rpm, with the supernatant reserved for enzyme-linked immunosorbent assay and molecular tests, including polymerase chain reaction (PCR). The sediment is then resuspended in 0.2–1 mL of RPMI for additional cytological and molecular analysis to identify malignant B or T cells.^[41,42]^

Although PPV may enhance visual acuity and alleviate symptoms, the high rates of false negatives necessitate multiple biopsies or even chorioretinal biopsies or subretinal aspiration for accurate diagnosis.^[43]^ Another potential complication of vitreous biopsy is tumor seeding through the sclerotomy port into the epibulbar space.^[44]^

#### Cytokine levels 

Both the cytokine profile in vitreous fluid and aqueous humor, including elevated levels of IL-10, an increased IL-10/IL-6 ratio, and interleukin score for IOL diagnosis (ISOLD), can serve as promising diagnostic tools to support the identification of VRL.^[45]^

Aqueous humor cytokine analysis effectively distinguishes VRL from uveitis, with VRL patients exhibiting significantly higher IL-10 levels and an IL-10/IL-6 ratio > 1.55, achieving 94.1% sensitivity and 100% specificity. After treatment with intravitreal methotrexate, improvements in BCVA were correlated with decreases in IL-10 and IL-6 levels.^[46,47,48]^ A cutoff of 65 pg/mL for IL-10 in vitreous fluid and 30 pg/mL in aqueous humor demonstrated 93% and 78% sensitivities, along with specificities of 100% and 97%, respectively. Furthermore, an IL-10/IL-6 ratio exceeding 1 in vitreous fluid exhibited a sensitivity of 93% and a specificity of 100%.^[49]^ Moreover, the random forest algorithm, utilizing IL-10 levels, demonstrated promising results for diagnosing VRL. This approach highlights the potential of machine learning to enhance diagnostic accuracy by identifying key immune mediators, suggesting that IL-10 could serve as a valuable biomarker in VRL diagnosis.^[50]^ Improvement in aqueous cytokine levels upon treatment indicates treatment response, with the mean time to normalize IL-10 levels being approximately 1.17 months. Consistently high IL-10 levels, particularly an IL-10 threshold of > 50 pg/mL, were associated with disease recurrence and relapse, suggesting that monitoring aqueous IL-10 levels can be a valuable tool for assessing treatment efficacy and detecting relapses in patients with PVRL.^[51,52]^

Elevated CSF levels of IL-10, independent of aqueous humor levels, were observed in 90.2% of VRL patients and were associated with intracranial lesions, suggesting that CSF IL-10 could be a valuable biomarker for monitoring disease progression.^[53]^

Combining cytologic smears with immunohistochemistry (IHC) and cytokine analysis significantly enhances the diagnostic accuracy for VRL, achieving a sensitivity of 92% and a specificity of 98%.^[54]^

#### Assessment of clonality through immunoglobulin gene rearrangement analysis

During normal B-cell development, the immunoglobulin heavy (IGH) and kappa light chain (IGK) genes rearrangements occur, enabling each B cell to produce a unique antibody. Neoplastic monoclonality of B- or T-lymphocytes in vitreous fluid can be identified through flow cytometry, IHC, and PCR to detect the CD20 population of B cells or to identify IGH or IGK gene rearrangements, provided that the vitreous specimen is sufficient for analysis.^[55,56]^

Studies showed that immunoglobulin clonality assays, particularly the IGK assay, demonstrate high specificity and sensitivity, making them valuable adjuncts in the comprehensive evaluation of patients suspected of having VRL. The sensitivity of standard clonality studies typically ranges from 46% to 95%, depending on the primer sets used.^[56,57,58,59,60,61,62]^ When both assays are combined, the overall sensitivity is further enhanced.^[63]^ IGK exhibited markedly higher specificity than IGH in diagnosing VRL and distinguishing it from uveitis (94.12% vs. 78.95% specificity). This is particularly relevant in cases of severe sub-RPE and retinal infiltration, where the IL-10-to-IL-6 ratio may deviate from typical patterns and warrants careful interpretation.^[64]^ Immunoglobulin gene repertoire analysis helps distinguish PVRL from PCNSL, with IGHV4-34 utilization markedly higher in PVRL (64%) than in PCNSL (34.7%), reflecting distinct clonal profiles.^[65]^

Studies revealed that combined assessment of cellular, cytokine, and proteomic biomarkers, including an increased IL-10/IL-6 ratio, elevated levels of IL-10, IL-1 receptor antagonist (IL-1 RA), monocyte chemotactic protein-1 (MCP-1), and macrophage inflammatory protein-1 β (MIP-1 β ), along with the presence of CD19+ B cells and atypical surface immunoglobulin light chain expression, exhibit strong diagnostic efficacy for VRL, especially in cases characterized by minimal cellular infiltration.^[66]^

#### Mutational analysis 

Recent studies have demonstrated that identifying recurrent mutations significantly enhances the diagnostic yield of vitreous specimens. Detecting the *MYD88* mutation may serve as a valuable adjunctive diagnostic tool for VRL.^[67,68]^ Both aqueous humor and vitreous fluids exhibited high sensitivity (67% and 75%, respectively) and specificity (100% for both samples), with the mutation becoming undetectable following treatment.^[69]^ Additionally, changes in cfDNA allele frequencies in post-treatment samples were found to correlate with interleukin-10 levels, confirming the link to treatment response.^[70]^

Despite the inherent challenges of low cellularity and high viscosity in vitreous and aqueous fluids, recent advancements in cell-free DNA (cfDNA) extraction^[71,72]^ and Next-Generation Sequencing (NGS)^[55,73,74]^ have significantly enhanced the ability to identify genomic mutations in VRL, particularly *MYD88* L265P,^[75]^ achieving sensitivity levels comparable to those observed with IL-10/IL-6 ratios.

*MYD88 and CD79B *mutations detected in cfDNA from CSF samples, in conjunction with elevated CSF IL-10 levels, may serve as promising adjunct tools to aid in diagnosing PVRL.^[76]^

Although CSF samples can assist in diagnosis, VRL exhibits distinct mutational profiles compared to PCNSL, notably featuring a higher prevalence of *MYD88* mutations and a lower frequency of IRF4 mutations in VRL.^[70]^

A recent systematic review shows that an IL-10/IL-6 ratio > 1 provides the highest sensitivity for diagnosing PVRL at 89.39%, with comparable sensitivity between vitreous and aqueous samples. While other diagnostic modalities, such as flow cytometry (88%) and PCR for monoclonal IgH rearrangements (85.1%), also show notable sensitivity, *MYD88* L265P and *CD79B* mutations are less effective, yielding sensitivities of 70% and 35.09%, respectively.^[56]^

Concise diagnostic criteria for VRL include integrated clinical manifestations, vitreous cytology, IHC, cytokine, gene rearrangement, and flow cytometry tests, with high sensitivity (97.5%) and specificity (100%).^[77,78]^

NGS has become a crucial tool for assessing clonality in VRL, markedly improving diagnostic accuracy over traditional methods. Unlike conventional techniques that separate amplification products by size, NGS relies on unique rearranged sequences, yielding superior results. Clonality testing using NGS can confirm a VRL diagnosis even with limited DNA samples, achieving nearly 100% accuracy in cases lacking cytological evidence.^[55,79,80,81]^

#### Emerging opportunities in diagnostic testing

Due to the difficulties associated with the cytological diagnosis of VRL, there is significant interest in exploring additional biological markers for the disease. In the last decade, numerous potential biomarkers have been studied, including microRNAs, B-cell regulatory cytokines, and various immunological proteins.^[82]^

Quantifying microRNAs has emerged as a promising approach for diagnosing VRL. Studies have shown that specific microRNAs, such as miR-155,^[83]^ miR-19b, miR-21, and miR-92,^[84]^ can differentiate lymphoma from uveitis. Notably, miR-92 demonstrated the highest accuracy in distinguishing lymphoma. Further research by Minezaki et al identified 293 differentially expressed miRNAs in vitreous samples, with miR-6513-3p showing the strongest discriminatory power.^[85]^ Researchers elucidated distinct microRNA profiles in extracellular vesicles derived from the vitreous and serum of patients with ocular sarcoidosis and VRL, indicating potential associations with disease pathogenesis and highlighting opportunities for developing novel biomarkers.^[86]^

The combination of vitreous cytokine profiling through logistic regression modeling^[87]^ and the assessment of altered serum microRNA expression patterns shows promise as a multifaceted biomarker approach to differentiate VRL from uveitis effectively.^[82,85]^ Overall, despite methodological differences, microRNA biomarkers offer a valuable direction for future VRL research.

Another group of potential biomarkers for VRL includes molecules that regulate B-cell activities, resulting in a unique proteomic profile found in the vitreous fluid of VRL patients. A comparative analysis of cytokine levels in the vitreous of VRL patients versus a control group showed significantly elevated levels of IL-22 in the VRL patients. Furthermore, the levels of IL-35 were identified as a strong predictor of poorer five-year survival outcomes ^[88]^

Aqueous humor levels of A proliferation-inducing ligand (APRIL) and B-cell activating factor (BAFF) serve as effective biomarkers for distinguishing VRL from uveitis while also reflecting therapeutic response during chemotherapy.^[89]^

### Imaging Modalities 

VRL presents diagnostic challenges due to its symptoms resembling uveitis, which can lead to delays in diagnosis and treatment, ultimately worsening patient outcomes. Although histopathological confirmation remains the diagnostic gold standard, multimodal imaging enhances early detection, guides timely intervention, facilitates disease monitoring, and provides critical prognostic information for visual and systemic outcomes.

#### Ultra-widefield color fundus photography (CFP)

Ultra-widefield CFP detects more frequent peripheral abnormalities compared to posterior pole findings in patients with VRL, aiding in early diagnosis of VRL.^[90]^ CFP enables direct visualization of vitreous haze, vitreous cellular debris, optic nerve edema,^[28,36]^ and cream-colored lesions corresponding to sub-retinal pigment epithelium (sub-RPE) lesions,^[91]^ as well as serous retinal detachments (SRDs)^[92,93]^ and vascular sheathing.^[94]^

CFP findings can significantly aid in predicting the prognosis of VRL. The presence of retinal or subretinal infiltrations is associated with a marked decline in visual acuity post-treatment.^[95]^ Pseudonecrotic retinal lesions (PRLs), observed in approximately 35.8% of eyes, serve as significant predictors of worse median BCVA outcomes. Specifically, eyes with PRLs show a BCVA of 2.4 logMAR (logarithm of the minimum angle of resolution), in contrast to 0.5 logMAR in cases without necrosis. PRLs are linked to complications such as optic disc swelling, retinal vasculitis, hemorrhage, and detachment, indicating a more aggressive clinical course and a worse visual prognosis.^[96]^ Additionally, patients presenting with vitritis tend to have a mean visual acuity of 20/34 at one year post-treatment. While those with optic disc edema or exudative retinal detachments often experience a severe reduction in vision, sometimes limited to "light perception," this highlights the importance of careful monitoring and assessment of these clinical features to better predict visual outcomes.^[97]^

In CFP, the vitreous haze may display uniform material with nonspecific characteristics, a "string of pearls" pattern (yellowish clusters of cells in lines) or an "aurora borealis" pattern (linear opacities with cells aligned along vitreous fibrils).^[98]^

#### Optical coherence tomography 

Optical coherence tomography (OCT) has become a valuable diagnostic tool for VRL, effectively detecting signs of lymphomatous infiltration even when vitritis limits the ability to perform a precise fundus examination. It enables the detection of characteristic features, such as vitreous cells in almost all VRL patients, as scattered hyperreflective foci in the posterior vitreous,^[15,99]^ outer retinal fuzzy borders,^[100]^ sub-RPE infiltrates with thickened RPE or RPE detachment,^[101]^ and hyperreflective subretinal infiltrates,^[102,103]^ and facilitates effective long-term monitoring of this rare and aggressive ocular malignancy. The characteristic OCT features of VRL, including preretinal deposits, intraretinal infiltrates, outer retinal atrophy, and RPE changes, in the absence of epiretinal membrane and central macular thickening, can reliably differentiate it from uveitis.^[104,105]^ OCT of VRL often reveals distinctive features, termed "vertical hyperreflective columns" (VHRCs), extending from the inner retinal layers to the RPE and are believed to correspond to microinfiltrates of malignant lymphoma cells.^[106]^ Pretreatment and recurrent VRL are more often characterized by subretinal or RPE abnormalities rather than primarily intraretinal changes.^[107]^ OCT analysis revealed that in eyes with observed tumor progression, there was an increase in intraretinal deposits compared to the initial presentation. Similarly, in eyes with tumor recurrence, there was a higher incidence of intraretinal deposits at the time of recurrence. Conversely, in eyes demonstrating tumor regression upon treatment, there were fewer vitreous opacities, intraretinal deposits, subretinal deposits, and sub-RPE deposits compared to the initial presentation.^[52,108,109,110]^ In the remission phase following intravitreal rituximab (IVR) treatment, there was a significant reduction in hyperreflective retinal dots (HRD), intraretinal infiltration, and outer retina fuzzy borders, along with decreases in central macular thickness (CMT) and choroidal thickness (CT),^[111]^ highlighting the decline in lymphoma-induced inflammation and infiltration.^[112]^

Chorioretinal atrophy (CRA) is a degenerative condition characterized by the loss of RPE and photoreceptors, often representing the end-stage of various vitreoretinal diseases. In patients with VRL, retinal infiltrates and macular involvement are significantly associated with an increased risk of developing CRA.^[113]^

OCT angiography has revealed a novel characteristic finding in VRL perivascular "flower-bud-like" lesions that correlate with sub-RPE changes.^[114]^ Swept-source optical coherence tomography angiography (SS-OCTA) demonstrates multiple vertical hyperreflective lesions in the retina, frequently associated with infiltrated retinal vessels. Notably, these lesions may diminish following treatment, suggesting their potential as biomarkers for VRL.^[115,116]^ Additionally, OCTA can identify hyperreflective infiltrates that exhibit hyperautofluorescence on fundus images, thereby enhancing the diagnostic accuracy for PVRL.^[117]^

#### Fundus autofluorescence (FAF)

FAF enables a noninvasive evaluation of retinal and RPE fluorophores. Hyperautofluorescence, observed in 61% of the cases, typically presents as granular patterns indicative of active PIOL. This phenomenon is associated with lymphomatous infiltration, leading to the accumulation of fluorophores. These hyperautofluorescent spots often correlate with nodular hyperreflective areas seen in OCT. Lymphomatous infiltration of the RPE as sub-RPE lesions may disrupt RPE metabolism, leading to hypoautofluorescence areas. Additionally, RPE atrophy following lesion regression is presented as hypoautofluorescent areas.^[118,119,121]^ Ultimately, extensive hypoautofluorescent areas and the absence of active/inactive regions are indicative of CRA.^[15,122]^ The leopard pattern had a 69% sensitivity and 100% specificity in detecting active lesions.^[19]^ FAF transitions from hyperautofluorescence to hypoautofluorescence following IVR treatment, indicating disease regression in VRL. This change highlights the effectiveness of FAF in monitoring treatment response and assessing disease progression in VRL.^[119]^

#### Fluorescein angiography

Changes in the RPE manifest as hypo- or hyperfluorescent round lesions with a "leopard spot" appearance in FA, especially during the late stages of examination, which are indicative of VRL. Neoplastic infiltrations appear as hypofluorescent areas because diffuse lymphomatous infiltrates beneath the RPE are impermeable to fluorescein, and tumor cell membranes act as a barrier that obstructs choroidal hyperfluorescence. Meanwhile, RPE atrophy manifests in hyperfluorescent regions. FA can reveal a gradually staining granular pattern of hypo- and hyperfluorescent areas corresponding to diffuse sub-RPE tumor infiltration without discernible nodularities. A mottled appearance in FA can often be observed, even in the absence of clinical disturbances in the RPE.^[123,124,125]^ The granular pattern of hypo- and hyperautofluorescence observed in FAF is reversed in FA, where hypofluorescent "leopard spot" lesions on FA correlate with hyperautofluorescent spots seen in FAF.^[126]^

One-third of patients show signs of inflammation on FA, including perivascular leakage, cystoid macular edema (CME), and optic disc leakage or staining, which are associated with optic disc infiltration and are indicative of potential CNS involvement. Vascular leakage indicates periphlebitis, which may result from inflammation or tumor cell infiltration of the vessel wall and typically resolves with appropriate treatment.^[122,127]^

A novel observation reported by Venkatesh et al in FA of PVRL, termed "capillary dropout," has been identified, suggesting potential retinal vascular occlusion due to malignant cells.^[128]^

While FA is a valuable tool for diagnosing VRL, its efficacy is significantly enhanced when coupled with other imaging modalities, such as OCT and ICGA. This multimodal approach facilitates a comprehensive assessment of the disease.

#### Indocyanine green angiography (ICGA)

ICGA displays round areas of focal hypocyanescence, typically located at the posterior pole and linked to sub-RPE infiltration. While ICGA identifies fewer lesions than FA and FAF, it helps exclude other conditions, such as white dot syndromes and sarcoidosis, and differentiate between VRL and choroidal lymphoma based on choroidal involvement.^[125,128,129]^

#### Ultrasound 

Vitreous opacity with homogenous hyperreflective corpuscular materials, posterior vitreous detachment, partial retinal detachment, widening of the optic nerve, elevated chorioretinal lesions, and irregular hypoechoic lesions beneath the retina are ultrasonography findings in VRL.^[19,130,131]^ B-scan ultrasonography offers distinguishing features of VRL compared to uveitis, including a lower incidence of vitreoretinal adhesion, a higher prevalence of retinal thickening or occupying lesions, and a notable centrifugal condensation pattern of vitreous haze, characterized by a peripherally hyperreflective appearance.^[132]^

Ultrasonographic elevated mass can aid in distinguishing VRL from other solid masses, such as uveal melanoma, metastatic carcinoma, and choroidal hemangioma. Additionally, the degree of corpuscular material seen on B-scan images is indicative of the severity of vitritis.^[133]^

Moreover, B-scan ultrasound is recommended for screening VRL over slit-lamp examination, as it offers more precise and more definitive diagnostic features.^[134]^

#### Anterior segment optical coherence tomography and in vivo confocal microscopy (IVCM)

While intermediate and posterior manifestations of VRL are well characterized, anterior segment involvement is also common. IVCM identifies various morphologies of keratic precipitates (KPs), including dendritic, nibbling, linear, globular, and stippled forms. Dendritic KPs, characterized by a hyperreflective core and pseudopodia, were found in 56.3% of cases.^[135]^ A unique floral pattern of KPs was observed in multiple patients, with complete and incomplete forms present.^[136]^ Alterations in KPs' morphology after chemotherapy can serve as valuable indicators of treatment efficacy.^[137,138]^

A recent study developed a machine learning model utilizing AS-OCT, which demonstrated an impressive diagnostic accuracy of 87% in effectively differentiating between VRL and vitritis in uveitis through radiomics and advanced machine learning techniques.^[139]^ Furthermore, AS-OCT has proven effective in monitoring the recurrence of VRL in the anterior vitreous and tracking potential ocular relapse by confirming the presence of cellular material and debris. The resolution of KPs serves as a critical indicator of therapeutic response in VRL.^[52,140]^

#### Electroretinography (ERG)

ERG enhances the evaluation of retinal dysfunction in VRL by detecting characteristic attenuation of a- and b-wave amplitudes, which localizes to areas of outer retinal layer pathology.^[141]^

### Risk of CNS Progression 

Approximately one-third of patients diagnosed with PVRL also exhibit concurrent PCNSL, and between 42% and 92% may develop PCNSL within an average period of 8 to 29 months following their initial diagnosis. Therefore, brain magnetic resonance imaging (MRI) should be considered for all patients suspected of having PVRL. Up to 90% of VRL patients had CNS lesions, primarily in forebrain tissues and outside the optic pathways and visual cortex, indicating multifocal involvement and the need for careful monitoring throughout their clinical course.^[142,143]^ In patients with suspected PVRL and negative MRI findings, cerebrospinal fluid (CSF) sampling is crucial for detecting CNS lymphoma, as 16.9% of such cases may present with CSF suspicious for lymphoma despite negative MRI results.^[144]^ Additionally, when MRI findings raise suspicion for CNS pathology but CSF cytology results are negative, an image-guided stereotactic brain biopsy should be performed.^[42]^ For follow-up, the patient should undergo MRI with contrast every three months for the first two years, transitioning to every six months thereafter, to monitor for the development or progression of CNS disease.^[145,146]^

Multivariate analyses revealed that B-cell clonality is a significant risk factor for CNS progression. In contrast, the completion of systemic high-dose methotrexate (HD-MTX) treatment was associated with a decreased risk of CNS progression and an enhancement in overall survival.^[147,148]^ The association between laterality and CNS progression remains heterogeneous across studies, with no consensus in the current literature.^[149]^

Additionally, in patients with PVRL, factors such as VRL recurrence, elevated IL-10 levels, and sub-RPE infiltration were associated with CNS involvement.^[150]^ The association between intraocular recurrence and CNS involvement is heterogeneous, with some studies suggesting that intraocular recurrence does not significantly impact overall survival or the likelihood of CNS relapse.^[151]^

Comprehensive genetic analysis identified ETV6 loss and PRDM1 alteration as potential predictors for CNS progression in PVRL, enabling the risk stratification model to guide personalized treatment.^[152]^

### Treatment Strategy 

There is currently no established gold standard for the treatment of VRL. Various intraocular and systemic therapies are available, but none have effectively eradicated the lymphoma or prevented its progression. In isolated VRL cases without CNS involvement, the treatment focuses on controlling the intraocular disease and preventing CNS complications. However, VRL spreads to the CNS in 60% to 90% of patients despite these efforts. Local therapies can manage intraocular symptoms but do not prevent CNS dissemination, prompting consideration of systemic prophylactic chemotherapy.^[153]^ De la Fuente et al reported a CNS dissemination rate of 37.5% with bilateral radiation therapy and systemic methotrexate,^[154]^ which is significantly lower than the 56–85% rate seen in other studies.^[155]^ The International Primary CNS Lymphoma Collaborative Group recommends high-dose systemic chemotherapy with intravitreal treatments for isolated VRL.^[156]^ However, Hashida et al cautioned that prophylactic systemic chemotherapy may only delay CNS involvement rather than prevent it entirely.^[157,158]^ A recent meta-analysis comparing ocular treatments, such as intravitreal methotrexate and radiotherapy, to systemic therapies like chemotherapy found that ocular treatment significantly reduced the risk of PCNSL (OR = 0.54, *P* = 0.02) and ocular relapse (OR = 0.26, *P* = 0.001) compared to systemic therapy. Notably, there were no significant differences in the progression to systemic disease or overall survival. Consequently, the addition of systemic therapy to ocular treatment provided no additional benefits for these patients.^[159]^

Local therapies for VRL include intravitreal injections of chemotherapeutic agents and ocular radiation therapy, with no clear preference for one over the other. Treatment choice should consider disease laterality and patient preferences. External-beam radiation therapy (EBRT) is recommended for patients without CNS involvement, delivering 35 to 40 Gray (Gy) in 15 fractions of 2 Gy each, with low rates of recurrence and radiation retinopathy; however, cataract is a common complication that can be surgically addressed.^[153,160]^ Intravitreal therapies, particularly methotrexate and rituximab, are also effective. The standard methotrexate regimen involves 400 μ g injected twice weekly for 4 weeks, then weekly for 8 weeks during consolidation, and monthly for 9 months during maintenance, showing low recurrence and minimal side effects like corneal epitheliopathy and transient intraocular pressure (IOP) increases. Rituximab, at 1 mg in 0.1 mL for 4 weeks, offers a valid alternative with less frequent injections and lower corneal toxicity risk while maintaining efficacy.^[161,162]^

In cases of CNS involvement, treatment typically involves an induction phase with HD-MTX, achieving remission rates of 72% for PCNSL and 94% for VRL. The International Extranodal Lymphoma Study Group-32 (IELSG32) identified the MATRix regimen, comprising methotrexate, cytarabine, thiotepa, and rituximab, as the standard for patients under 70 years old.^[163,164]^ While this regimen can be combined with whole brain radiation therapy, it is associated with a significant rate of adverse events, and about 60% of patients achieve a complete response after induction. However, consolidation therapy is crucial to reduce relapse risk, with options including whole brain radiation, additional systemic chemotherapy, or autologous stem cell transplantation.^[164,165]^ In cases of PCNSL relapse, re-administration of HD-MTX may be considered if the initial treatment was effective.^[166]^ Other treatment alternatives include thiotepa-based chemotherapy followed by autologous stem cell transplantation, intrathecal cytarabine, and combinations of high-dose cytarabine with pemetrexed, lenalidomide, pomalidomide, or ibrutinib. Notably, lenalidomide and temozolomide have shown promise as oral therapies, warranting further investigation as potential first-line treatments. Ibrutinib, which inhibits Bruton's tyrosine kinase involved in B-cell receptor signaling and lymphoma growth, has also demonstrated clinical activity in relapsed and refractory cases of PCNSL and VRL.^[167,168]^ A recent systematic review compared monotherapy, such as Bruton's tyrosine kinase inhibitors, to combination therapies primarily using intraocular methotrexate (ioMTX) with chemotherapy or radiotherapy. Combination therapies demonstrated significantly higher overall response rates (96% vs. 72%) and complete response rates (92% vs. 63%), as well as longer median progression-free survival (28.8 months vs. 13 months, *P* = 0.012). However, they also had a higher incidence of severe side effects (45% vs. 8%). While BTK inhibitors were well-tolerated, their long-term effectiveness and the need for further studies on monotherapy for VRL were emphasized.^[169]^

### Prognosis and Survival Rate 

VRLs are highly aggressive malignancies with a significant risk of recurrence. Patients concurrently diagnosed with CNS lymphoma and VRL had significantly shorter overall survival and a lower 5-year survival rate compared to those with PVRL alone and those with VRL after primary CNS diagnosis.^[150]^ Current data indicate a median overall survival of approximately 33 months and a median progression-free survival of only 11 months for VRL patients. The overall 5-year survival rate remains low, at < 25%, reflecting the aggressive nature of the disease and its propensity for recurrence.^[170]^

In contrast to the poor prognosis associated with cerebral relapses, intraocular recurrence in VRL does not significantly compromise overall survival or visual acuity compared to nonrecurrence. Factors such as younger age, isolated PVRL without CNS involvement, elevated IL-10, and no prior intravitreal chemotherapy may predict an increased risk of intraocular recurrence.^[151]^

In patients with VRL, worse baseline visual acuity and retinal or sub-RPE infiltrates have been identified as predictive factors for a shorter survival time and poor visual prognosis in patients with VRL.^[171,172,173]^ PVRL is associated with the highest retinal infiltration and the worst visual acuity at diagnosis, while secondary VRL patients experience a shorter diagnostic delay.^[174]^

High intravitreal levels of IL-35 are linked to poor prognosis in patients with B-cell VRL, with a 5-year overall survival rate of 40% for those with elevated IL-35 compared to 83.3% for those with lower levels (*P* = 0.024).^[175]^ Patients with IgA levels below 184 mg/dL experienced a lower three-year survival rate (*P* = 0.03) and more frequent recurrences (3.2 vs. 1.8 times, *P* = 0.02).^[176]^

The IELSG has identified five clinical variables that correlate with the prognosis of PCNSL. These factors include elevated lactate dehydrogenase, age over 60, and an Eastern Cooperative Oncology Group (ECOG) performance status > 1, as well as elevated CSF protein and tumor location in deep brain regions. Survival rates significantly vary based on the number of adverse risk factors present, with rates of 80% for 0–1 factors, 48% for 2–3 factors, and 15% for 4–5 factors.^[177]^

### Uveal Lymphoma 

Primary uveal lymphomas originate in the choroid, iris, or ciliary body, distinct entities from VRLs in terms of etiopathology, and are not linked to CNS lymphoma. Uveal lymphoma commonly affects older male patients who present with blurred vision or metamorphopsia.^[178]^

### Choroidal Lymphoma 

Choroidal lymphoma is the most commonly observed primary uveal lymphoma and secondary IOL.^[179,180]^ Primary choroidal lymphomas are primarily indolent, low-grade B-cell neoplasms that are histologically classified as EMZL. They have historically been referred to as “uveal pseudotumors” or lymphoid hyperplasia due to their low-grade histological characteristics and minimal metastatic potential.^[178]^

Compared to primary choroidal lymphoma, secondary cases were characterized by a shorter duration of ocular symptoms, less prior steroid use, and more frequent bilateral ocular involvement. Eyes with secondary choroidal lymphoma also had a higher rate of poorer visual outcomes and more extensive intraocular involvement, including the iris, ciliary body, and vitreous. Morphologically, secondary choroidal lymphoma was more likely to be high-grade, in contrast to the predominantly low-grade nature of primary cases. Significantly, none of the patients with primary choroidal lymphoma developed systemic disease during the follow-up period. The most common types of primary choroidal lymphoma and systemic lymphoma associated with secondary choroidal lymphoma are extranodal marginal zone B-cell lymphoma and diffuse large cell lymphoma, respectively.^[181]^

Choroidal lymphoma is typically diagnosed through a comprehensive ophthalmological assessment, including ophthalmoscopy, ultrasonography, fluorescein angiography, indocyanine green angiography, and MRI.^[129,181,182]^ B-scan ultrasonography of choroidal lymphoma reveals diffuse choroidal thickening, extrascleral extension, and crescentic thickening with subsequent conjunctival salmon patch, while funduscopic examination demonstrates yellow-white infiltrates.^[92,178,183]^ Importantly, enhanced depth imaging optical coherence tomography (EDI-OCT) has emerged as a valuable tool, as it can depict subtle choroidal thickening and characteristic surface topography patterns that correlate with the extent of lymphomatous infiltration, a placid appearance with thin infiltration (average 1.7 mm), a rippled appearance with moderate infiltration (average 2.8 mm), and a "seasick" appearance with thick lymphomatous infiltration (average 4.1 mm).^[184]^

Using ICGA and OCT demonstrates a valuable diagnostic approach for evaluating choroidal lymphoma. ICGA can reveal discrete areas of choroidal hypopigmentation, while OCT can detect diffuse infiltration of the choroid. The discrepancy between these imaging findings may serve as a distinctive diagnostic characteristic of choroidal lymphoma.^[185]^

Ocular fluid sampling provides a low-morbidity approach to confirm suspected choroidal lymphoma with intraocular fluid cellularity, in which clonal B-cells support diagnosis.^[186]^

Hyper-reflective bands in the subretinal space and infiltrates between the RPE and Bruch's membrane can help differentiate PVRL from choroidal lymphoma.^[102]^

While definitive diagnosis of choroidal lymphoma ultimately requires biopsy,^[187]^ flow cytometry, PCR,^[188]^ and immunohistochemical staining for B-cells and monoclonality^[189]^ can provide valuable supplementary information to confirm the diagnosis.

### Ciliary Body Lymphoma 

Ciliary body lymphoma is often associated with blurred vision, red eye, and eye pain, as well as ciliary body injection, anterior chamber reaction, pseudohypopyon, and posterior synechiae in the clinical examination. The symptoms and clinical findings of ciliary body lymphoma often overlap with anterior uveitis. However, an atypical anterior chamber reaction and ciliary body lymphoid infiltration can help differentiate the conditions. Unfortunately, aqueous samples are frequently non-confirmatory due to low cellularity, so vitreous biopsy should be considered.^[190,191]^

Ultrasound biomicroscopy (UBM) examination reveals a 360-degree, ring-like, solid infiltration of the ciliary body with low, homogeneous internal reflectivity, warranting the term "ring lymphoma.” Due to the rarity of these lesions, differentiating them from other ciliary body tumors, such as melanoma, can be challenging. While clinical features and UBM acoustic characteristics may aid diagnosis, overlapping presentations can complicate the distinction, as some ciliary body melanomas also show low to medium reflectivity with irregular features.^[192]^ Distinctive UBM characteristics of ciliary body melanomas, such as irregular acoustic sites, cystic spaces, and hollowness, can help differentiate them from the more homogeneous presentation of lymphoma.^[193]^ However, a definite diagnosis and distinction are made by histological analysis.

### Iris Lymphoma 

Iris lymphomas are typically unilateral secondary manifestations of aggressive, high-grade B-cell non-Hodgkin lymphoma. The overlapping clinical presentation with anterior uveitis, including blurred vision, elevated IOP, KPs, and anterior segment inflammation, can complicate diagnosis and lead to delays. Although the presence of a whitish iris mass, abnormal iris vessels, pseudohypopyon, and hyphema may help distinguish iris lymphoma, definitive diagnosis ultimately requires tissue biopsy or aqueous cytology.^[194,195]^

UBM and B-scan ultrasonography can be valuable diagnostic tools in cases where iris thickening is suspected, mainly when the media is hazy, obscuring a clear view.^[196]^

Secondary glaucoma is a common complication of iris lymphoma, occurring in up to 29% of cases. The mechanisms underlying this secondary glaucoma include angle invasion, angle closure, and hyphema.^[197,198]^

### Treatment Strategy 

Management of primary uveal lymphoma typically involves a multidisciplinary tumor board due to its rarity. EBRT, with doses ranging from 40 to 80 Gy, is a standard treatment option that often results in complete and durable remission while being well tolerated by patients.^[199]^ Prognosis is generally favorable, especially for cases classified as EMZL, with studies showing 5-year survival rates of 100% and complete remission rates of up to 79% with treatments such as EBRT or rituximab.^[200]^ However, the lack of established guidelines for primary iris or ciliary body lymphoma complicates treatment decisions and prognosis predictions. In elderly patients with slowly progressive disease, observation may be a viable management strategy.^[178]^ Untreated primary uveal lymphoma can lead to severe complications, including glaucoma and vision loss, which may necessitate enucleation. Therefore, close monitoring and annual evaluations are recommended for relapse or systemic lymphoma, despite the rarity of dissemination.^[194]^ Post-treatment monitoring for iris lymphoma involves regular ophthalmic follow-ups every 6 weeks for the first 3 months and quarterly for the next 3 years, focusing on visual acuity, IOP, and imaging studies. Hematology check-ups are scheduled every 3 to 6 months, with re-evaluation by the tumor board if recurrence is suspected.^[201]^

### Prognosis 

Uveal lymphoma is predominantly low-grade and less aggressive compared to other lymphoma types. Overall, patients experience favorable outcomes, with a complete remission rate of 78.6% after a median follow-up of 30.3 months and an impressive 5-year survival rate of 100%. Notably, all patients with localized disease treated with EBRT at a median dose of 3060 centigray (cGy) achieved complete remission, with similar success reported at doses of 3500 cGy. Additionally, some patients attained stable or partial remission, particularly those receiving rituximab, though distant relapse rates of 50% have been noted with rituximab monotherapy. Importantly, there have been no lymphoma-related fatalities among the cohorts studied, although the small sample size limits comprehensive analysis of treatment outcomes based on therapy selection.^[178,181,202]^

### Secondary Intraocular Lymphoma (SIOL)

SIOLs originate outside the CNS and subsequently metastasize to intraocular tissues. Regarding hematogenous dissemination, the choroid is the most commonly affected site.^[180]^ Involvement of the iris and ciliary body is less frequent,^[194]^ and metastasis of systemic lymphoma to vitreoretinal structures is extremely rare.^[6]^ Most intraocular metastatic lymphomas, similar to systemic lymphomas, are of B-cell origin, with DLBCL being the most common subtype.^[203]^

SIOLs typically present as pale choroidal lesions with associated subretinal fluid resembling mottled brown spots. In iris involvement, anterior chamber cells and KPs are frequently observed.^[18]^

Diagnosis of SIOLs is suspected in patients with a history of systemic lymphoma and confirmed through histopathologic examination.^[204]^ Management usually involves systemic therapy, potentially with intravitreal chemotherapy, and may require enucleation due to extensive disease.^[205]^ SIOL often indicates advanced metastatic disease, with prognosis generally being poor and dependent on the primary malignancy.^[206]^

**Table 1:** Subtypes of intraocular lymphomas

**Variables**		**Primary vitreoretinal lymphoma**	**Primary uveal lymphoma**
Epidemiology		F > M aged 50–70 years	M > F Mean age > 60 years
Histology		Primarily diffuse large B-cell lymphoma; rare cases of T-cell or NK-cell origin.	Primarily low-grade extranodal marginal zone lymphoma; may also include high-grade B-lymphocytic neoplasms in rare cases of iris lymphoma.
CNS involvement		Present in 16–34% at diagnosis. Up to 90% develop CNS lymphoma within 1 year.	No association with CNS lymphoma; distinct entity from vitreoretinal lymphomas.
Ophthalmic findings	Ultra-widefield color fundus photography	Vitreous haze, optic nerve edema, cream-colored lesions, and serous retinal detachments. Presence of pseudonecrotic retinal lesions.	Yellow-white infiltrates in choroidal lymphoma; may show salmon-patch lesions due to scleral infiltration. Iris lymphoma may present with a whitish iris mass, abnormal iris vessels, keratic precipitates, and hyphema. Ciliary body lymphoma often shows signs of anterior chamber reaction and pseudohypopyon, resembling anterior uveitis.
	Optical coherence tomography	Vitreous cells, sub-RPE infiltrates, and hyperreflective subretinal infiltrates. Features like "vertical hyperreflective columns" indicate microinfiltrates. Effective in monitoring disease progression and treatment response.	Enhanced depth imaging shows subtle choroidal thickening and surface topography patterns (placid, rippled, seasick appearances) in choroidal lymphoma. Useful for assessing iris thickening and anterior chamber reaction.
	Fundus autofluorescence	Hyperautofluorescence indicates active disease. Patterns correlate with nodular areas on OCT; hypoautofluorescence suggests RPE atrophy or CRA.	
	Fluorescein angiography	Hypo- and hyperfluorescent lesions with a "leopard spot" appearance. Granular staining patterns indicate sub-RPE infiltrates.	Limited diagnostic benefit; not typically used for uveal lymphoma diagnosis.
	Indocyanine green angiography	Hypocyanescent lesions linked to sub-RPE infiltration.	Discrete areas of choroidal hypopigmentation in choroidal lymphoma; not typically used for ciliary body or iris lymphomas.
	Ultrasound	Vitreous opacity, retinal detachment, and elevated lesions.	Choroidal lymphoma demonstrates diffuse choroidal thickening, extrascleral extension, and crescentic thickening. Ciliary body lymphoma shows a 360-degree, ring-like solid infiltration with low, homogeneous internal reflectivity ("ring lymphoma"). UBM is useful for detecting iris thickening, especially when media is hazy.
Diagnostics		Vitreous sampling via pars plana vitrectomy and aqueous humor. Cytology, immunophenotyping for B-cell markers, cytokine analysis of IL-10 and IL-6, and PCR for clonality assessment. Mutational analysis detects *MYD88* mutations. Future research focusing on microRNA and B-cell regulatory cytokines.	Definitive diagnosis through biopsy to identify malignant cells; less invasive options (epibulbar, conjunctival, anterior chamber) may yield reactive cells, while choroidal biopsy is needed for cytopathologic diagnosis. Flow cytometry and immunohistochemistry confirm B-cell lineage and monoclonality.
Management		Local intravitreal chemotherapy (often methotrexate-based) and ocular radiation, as well as systemic chemotherapy, particularly high-dose methotrexate, with rituximab.	Nonstandardized due to rarity; external beam radiotherapy is effective for primary choroidal lymphoma, with excellent prognosis (5-year survival ∼ 100%). Close follow-up recommended to prevent complications like glaucoma and vision loss.
CNS, central nervous system; RPE, retinal pigment epithelium; OCT, optical coherence tomography; CRA, chorioretinal atrophy; PCR, polymerase chain reaction.			

### Intravascular Large B-cell Lymphoma

Intravascular large B-cell lymphoma is a rare and aggressive hematological malignancy characterized by the proliferation of large B-cells within blood vessels, often sparing surrounding tissues.^[207]^ Ocular involvement is often bilateral (84% of cases). About 60% of patients exhibit detectable intraocular symptoms, while 24% present with extraocular signs, including diplopia, ptosis, and ophthalmoplegia. Common intraocular findings include SRD (46%), retinal hemorrhages (32%), and changes in the RPE. Other ocular manifestations may consist of choroidal thickening, vitritis, cotton-wool spots, and subretinal lesions. The condition has a slightly higher incidence in males, with a male-to-female ratio of 1.08:1, and the average age of diagnosis is 65.1 years (ranging from 38 to 82 years).^[208,209]^

Fundoscopic examinations often reveal multifocal SRD, Roth spots, and cotton wool spots. EDI–OCT is particularly useful for identifying choroidal infiltration, which is marked by the loss of distinct choroidal features. This imaging technique has also revealed changes in the RPE, the presence of subretinal fluid, choroidal thickening, and detachment of the bacillary layer.^[210]^ FAF imaging gives information about RPE condition, with hyperautofluorescent areas often associated with SRF and hypoautofluorescent regions indicating potential atrophy. FA is widely utilized to detect pinpoint hyperfluorescent leakage, subretinal pooling, and signs of choroidal ischemia. ICGA provides additional information about choroidal circulation, frequently highlighting hypofluorescent spots that suggest underlying abnormalities. B-scan ultrasound is effective in assessing structural changes, such as SRD and diffuse choroidal thickening.^[208,209]^

Diagnosis typically requires a tissue biopsy. Timely treatment with systemic chemotherapy and rituximab has been shown to significantly improve ocular symptoms, with 62% of patients experiencing nearly complete recovery.^[208,211]^

##  CONCLUSION 

In conclusion, IOLs often masquerade as intraocular inflammation, leading to misdiagnosis, delayed treatment, and subsequently increased mortality rates. A clear consensus on diagnosis and management still needs to be discovered by ophthalmologists and oncologists-hematologists. Patients with recurrent uveitis unresponsive to steroids should be referred to specialized centers for accurate diagnosis. Comprehensive multimodal imaging techniques, including FA, OCT, and ICGA, are crucial for identifying IOL features, particularly in cases of prolonged posterior uveitis [Table 1]. While cytologic analysis and IHC are key diagnostic methods, their low sensitivity warrants additional molecular assessments, such as detecting the *MYD88* L265P mutation and analyzing the levels of IL-10 and IL-6. Upon diagnosis of VRL, evaluating patients for neurological symptoms in consultation with an oncologist is essential, along with neuroimaging, to assess CNS involvement. The diagnosis and prognosis of IOL are expected to improve in the future due to advancements in less invasive molecular diagnostic techniques and increased awareness among healthcare providers. Clinicians should exercise caution in evaluating all patients with potential IOL and promptly refer them to oncologic specialists for expedited assessment and treatment.

##  AI Disclosure

During the preparation of this work, the authors used DeepSeek (DeepSeek, Version 2) for minor language editing and proofreading to improve clarity and readability. All scientific content, data interpretation, and conclusions remain solely the work of the human authors.

##  Financial Support and Sponsorship

None.

##  Conflicts of Interest

None.
